# Cystotome Use for Impassable Biliopancreatic Strictures: Efficacy and Safety—Systematic Review and Single Center Case Series

**DOI:** 10.14309/ctg.0000000000001019

**Published:** 2026-03-17

**Authors:** Gabriel Marcellier, Nathaniel Edery, Carlos Pratico, Benedicte Jais, Alain Berson, Frederique Maire, Paul Rivallin, Frederic Prat

**Affiliations:** 1 Endoscopy Unit, Beaujon Hospital, Clichy, France;; 2 Endoscopy Unit, Armand Brillard Hospital, Nogent-Sur-Marne, France;; 3 Pancreatology, Beaujon Hospital, Clichy, France.

**Keywords:** cholangiopancreatography, biliary stricture, pancreatic stricture

## Abstract

**INTRODUCTION:**

Certain biliary or pancreatic duct strictures can be crossed with a guidewire but remain impassable by standard dilation devices. Alternative strategies are sometimes required, such as drilling into the stricture with a cystotome, a 6Fr wire-guided electrocautery catheter. Data on the efficacy and safety of this rescue technique are scarce. We conducted a systematic literature review complemented by our single-center experience using a cystotome for unpassable biliopancreatic strictures.

**METHODS:**

A systematic review of PubMed, Embase, Google Scholar, and Cochrane library was performed, including studies using a cystotome for biliary or pancreatic strictures that could not be dilated with standard accessories. Alternative devices (e.g., Soehendra) were excluded. Primary outcomes were the combination of technical success (ability to traverse the stricture and deploy a stent) and safety (absence of procedure-related adverse event). Clinical success (short- and long-term) were collected if available. Our review was supplemented with consecutive cases performed at Beaujon Hospital (Clichy, France) from 2020 to 2025.

**RESULTS:**

A total of 102 patients were included (93 patients from 9 studies and 9 from our center). The primary composite outcome, combining technical success and safety, was achieved in 90.2% of patients. Technical success was 100% (102/102), with clinical success reported in 94% (84/89). Cystotome-related mild adverse events occurred in 9.8% of patients.

**DISCUSSION:**

Cystotome-assisted electrosurgical drilling seems to be technically reliable and effective for complex biliopancreatic strictures that are resistant to standard dilation. This comprehensive study could encourage to integrate the cystotome in the endoscopists' endoscopic retrograde cholangiopancreatography toolbox.

## Graphical abstract

**Figure:**
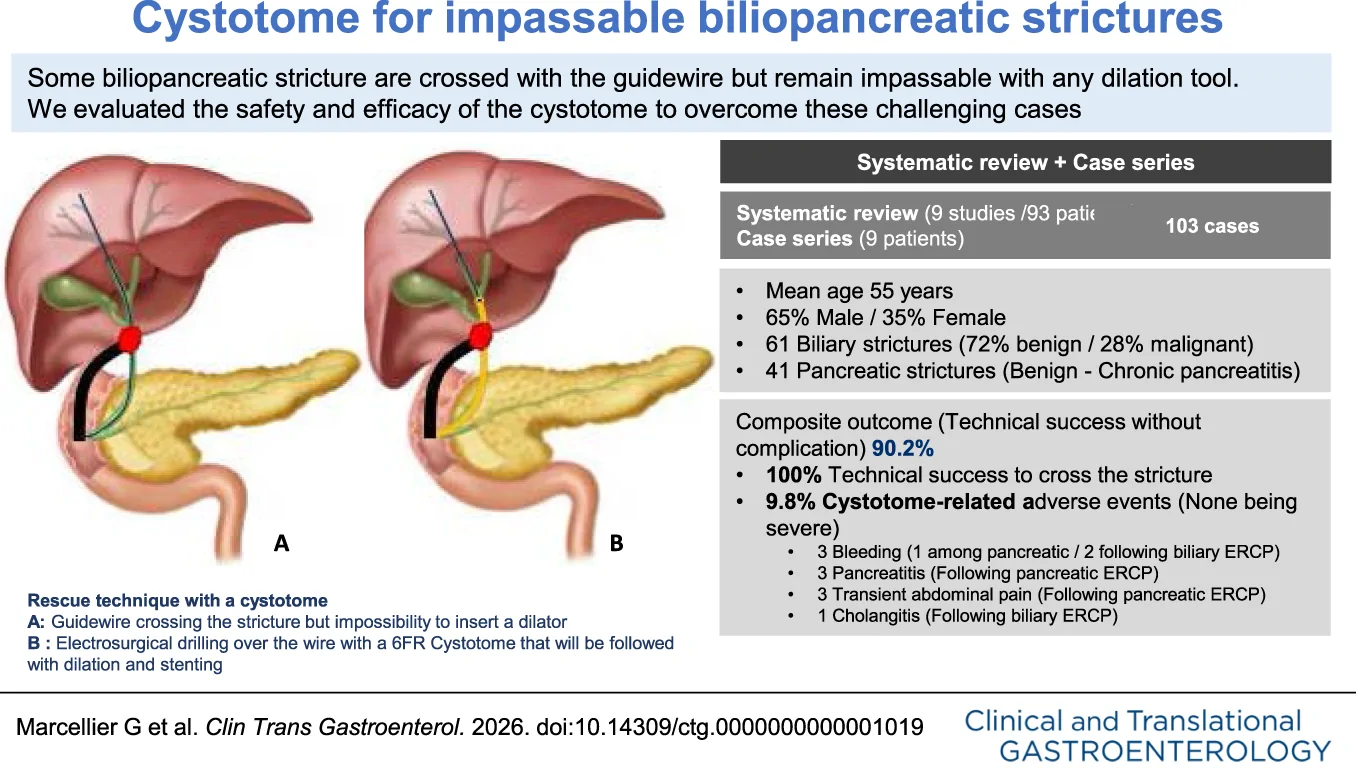

## INTRODUCTION

Strictures of the biliary or pancreatic ducts account for a substantial share of endoscopic retrograde cholangiopancreatography (ERCP) cases.

Whether biliopancreatic obstruction is caused by malignant (1) (pancreatic and hepatobiliary carcinomas, liver metastasis or other) or benign conditions (2) (e.g., postliver transplant anastomotic strictures, biliary injury, chronic pancreatitis, sclerosing cholangitis…), ERCP remains the mainstay of biliary drainage.

However, achieving functional and sustained biliary drainage by using the anatomic, retrograde route can be challenging when a stricture is long, angulated, tortuous or especially tight and fibrous. In these cases, despite achieving the critical step of passing a guidewire across the stricture, it may occur that dilating and stenting the obstacle remains impossible.

To overcome this obstacle, some daring endoscopists have used incisional devices to drill a passage through the stricture. One such tool is the small-caliber cystotome, originally conceived as a coaxial electrosurgical tool designed to create a transmural tract during endoscopic ultrasound (EUS)-guided interventions such as pancreatic fluid collection drainage or hepaticogastrostomy (3). After guidewire passage, cystotome-assisted electrosurgical cutting can be used to create a tract across the stricture when standard devices fail, enabling subsequent dilation and stent deployment.

By searching the literature, we found that although several teams published case series of biliopancreatic stricture management using a cystotome, no large study to date has investigated the safety and efficacy of this technique that remains confined to mostly anecdotal use in expert centers.

We therefore undertook the first systematic review investigating the safety and efficacy of cystotome drilling to pass severe biliopancreatic strictures. Our literature analysis was completed with our own case series.

## METHODS AND LITERATURE SEARCH

### Literature screening

According to the PRISMA 2021 guidelines (4), a systematic search was conducted in PubMed, Embase, Google scholar databases and the Cochrane Library with no language or date restrictions. Databases were last searched on February 23, 2026.

Search strategies combined the following keyword combinations: “cystotome” OR “diathermic dilator” AND “biliary stricture” OR “pancreatic stricture”. Complete search strategy for each database with the number of publications identified is described in Supplementary Appendix 1 (see Supplementary Digital Content, http://links.lww.com/CTG/B491).

Studies were eligible if they enrolled pediatric or adult patients with benign or malignant strictures of the biliary tree and pancreatic duct. Cases of digestive stricture were excluded. We focused exclusively on cystotome-assisted recanalization after failure to create a path with standard dilators. Reports describing the use of other devices (e.g. Soehendra stent retriever, needle knife, or wire-guided snare forceps) were excluded from the review. If a study presented cases with the use of different techniques, only patients in whom a cystotome was used were included. After defining the search strategy for each database, 2 independent investigators (G.M. and N.E.) conducted the literature search and screened the retrieved records. Studies were assessed sequentially based on title, abstract, and full-text review to determine eligibility. In cases of disagreement regarding study inclusion, discrepancies were resolved through discussion and consensus with a senior investigator (F.P.). No restriction was placed on study design: prospective or retrospective cohorts, case series, and single case reports were all eligible, provided that data for 1 of our primary outcomes could be extracted. Grey literature (abstracts/videos) was included when sufficient information was available to address the primary outcome measure.

Literature screening was completed with a single-center case series of the patients whose complex stricture required using a cystotome. All consecutive cases performed by a single senior operator (F.P.) at Beaujon Hospital (Paris Public Hospital Network, Clichy, France) from 2020 to 2025 were included in the study. The same outcomes as for the literature review were applied and the cases were included in the statistical analysis. This retrospective observational study complied with French regulations (CNIL MR-004). Patients were provided the opportunity to object to the use of their data (nonopposition procedure). As the study involved anonymized data collected during routine care without intervention, formal IRB approval was not required.

The typical procedure description includes the use of a duodenoscope under general anesthesia to perform ERCP. The stricture must have been passed with a 0.025” or 0.035” guidewire (we used either 0.025” visiglide2 [Olympus, Japan] or 0.035” hydrophilic Stiff Terumo [Terumo, Japan]) under fluoroscopic control after contrast injection from below has offered a clear depiction of the ducts beyond the stricture and confirmed the correct position of the wire, to safely proceed. When repeated efforts to insert a regular hydrostatic dilator through the stricture have failed, a 6FR Cystotome (Endo-Flex, Medi-globe, Germany) is advanced over the wire under fluoroscopic control applying either a pure cut or a sequential cut/coagulation (i.e., Endocut on VIO 200 or VIO3 surgical units, ERBE, Germany) current until the cystotome passes the stricture effortlessly by. A balloon dilator can usually be positioned afterward to proceed with subsequent steps, including dilation, cytology sampling when needed, and stent placement. Figure 1 depicts a 6Fr Cystotome.

**Figure 1.**
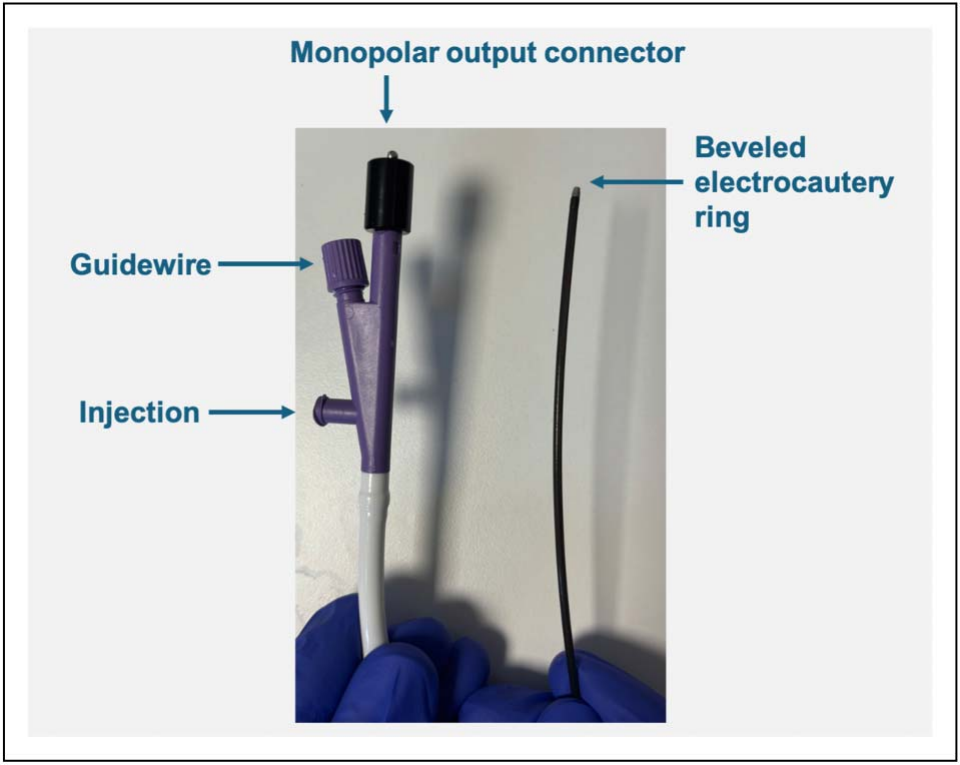
6Fr cystotome (Endo-Flex, Medi-globe, Germany).

### Outcomes

The aim of this study was to assess the cystotome’s ability to overcome reluctant strictures without complication, not to demonstrate superiority when compared with alternative techniques. The main outcome measure was therefore the combined ability to technically perform the drainage and to achieve it uneventfully.

- Technical success was defined as the ability of the cystotome to create a tract across the stricture, thereby permitting completion of the planned therapy—dilation, and/or stent deployment.
- Procedure-related adverse events (AEs) were graded using the AGREE classification, applying Clavien-Dindo classification to endoscopy (5,6), whenever possible (adverse event are severe if AGREE ≥ 3). AGREE classification was retained because it provides a standardized, treatment-based severity scale that can be uniformly applied across different types of complications and allows reproducible grading, even when detailed severity descriptors are not explicitly specified in the original reports. Causality between the cystotome itself and the complication is difficult to assess. Unless otherwise specified, any adverse event (such as bleeding or pancreatitis) was attributed to the cystotome by default. Conversely, if an event such as bleeding or perforation was directly related to another part procedure (sphincterotomy for instance), this was reported but not attributed to the cystotome, and therefore not affecting the primary end point.
- The main outcome was reached if technical success was achieved without procedure-related adverse events.

Secondary outcomes included clinical success, defined as a subsequent improvement or resolution of the stricture-related clinical condition that initially motivated the procedure. This definition was widely adopted among studies but there was a strong heterogeneity regarding the timing of clinical assessment. We decided clinical success to be achieved when clinical improvement within 1 week was reported and to report separately any long-term outcome (>3 months) when available.

In addition to those end points, several descriptive parameters were analyzed whenever reported in the source articles: patients characteristics (age, sex, indication of the procedure, stricture characteristics).

### Statistical analysis

Statistical analysis was performed with R-Software V4.4.2. Weighted mean and standard deviation were calculated. Descriptive statistics were used to summarize patient demographics, stricture characteristics, and procedural details. Clinical and technical success rates were calculated as well as adverse events rates. Risk of bias was assessed for each study by using the JBI Critical Appraisal Checklist (7).

No formal meta-analytic model was applied, given the limited number of studies, the heterogeneity in outcome definitions and follow-up durations, and the predominance of retrospective case series and reports.

## RESULTS

### Systematic review

A total of 96 studies were initially identified using our search strategy (Figure 2): 61 were excluded for being off the mark. One study was excluded for including only digestive strictures, 3 described the use of noncystotome devices, 1 was a literature review, 2 were case reports but were shortly followed by case series from the same author with probable duplicate, 1 reportedly used a cystotome to repermeabilize a stent but not to pass a stricture and 1 used a nonwire-guided cystotome percutaneously. Seventeen were found to be duplicates across databases. A total of 93 patients from 9 studies were identified from the systematic review. The final population including our case series included 102 patients. There were no discrepancies between G.M. and N.E. regarding study selection. All included studies complied with the JBI critical appraisal checklist (see Supplementary Appendix 2, Supplementary Digital Content, http://links.lww.com/CTG/B491 for further information). One case series was included despite the lack of standardized inclusion criteria, and 1 series was included based on a congress abstract with fewer information available.

**Figure 2.**
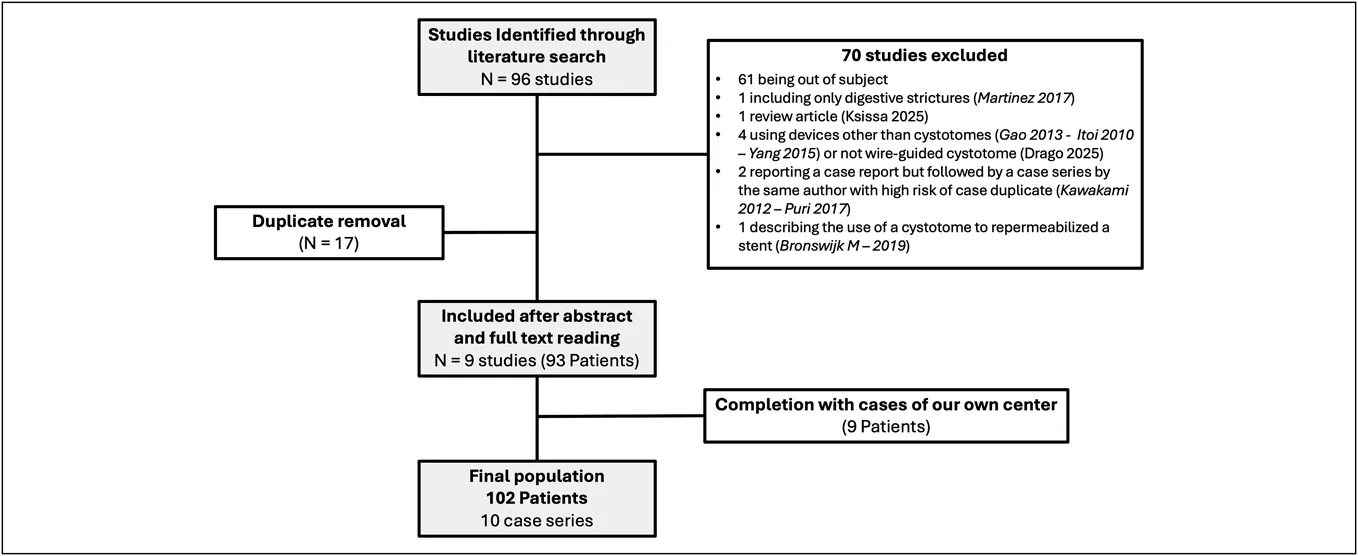
Prisma flow chart of the study.

### Characteristics of the study population

A total of 102 patients were included in the analysis, 9 from our case series, 93 from literature screening (8–16). Patients' demographics and outcomes are reported in Table 1.

**Table 1.** Included studies

Study	Design	Mean age (yr)	Sex	Stricture location and indication	Patients (N)	Main outcome—procedural success		Secondary outcome	
						Technical success (%)	Adverse event rate N (%)	Clinical success (%)	Long-term outcome if available
Kawakami et al (8)	Case series	62	12M 10W	Biliary duct (16) • 3 benign (PO) • 13 malignant (CCK—liver metastasis) Pancreatic duct (6) • 6 benign (CP)	22	100	2 (9.1%) • 1 hemobilia on a CCK • 1 pancreatitis after pancreatic duct treatment (mild) • No SAEs	100	NA
Puri et al (9)	Case series	45	14M 11F	Biliary duct (19) • 19 benign (PO) Pancreatic duct (6) • 6 benign (CP)	25	100	0 (0%)	100	7/19 biliary strictures persistent at 6 mo One-sixth pancreatic stricture persistent at 6 mo
Kato et al (10)	Case series	63	8M 5F	Biliary duct (13) • 13 benign (9 PO—3 ischemic—1 inflammatory stricture)	13	100	2 (15.4) • 1 hemobilia • 1 cholangitis • No SAEs	NA	62% were stent free at the end of follow-up (minimum 405 d) 5 (42%) hat a relapse during follow-up (33–964) d
Rana et al (11)	Case series	NA	8M 1F	Pancreatic duct (9) • 9 benign (CP)	9	100	3 (33.3%) • 3 transient abdominal pain • No SAEs	89	NA
Sundaram et al (12)	Case series	30.8	8M 2W	Pancreatic duct (10) • 10 benign (CP)	10	100	2 (20%) • 1 pancreatitis (mild) • 1 bleeding • No SAEs	88	No relapse 3 mo of follow-up
Tringali et al (13)	Case series	44.5	2M	Biliary duct (2) • 2 benign (PO)	2	100	0 (0%)	100	No relapse At 9-mo and 12-mo follow-up respectively
Lee et al (14)	Case series	68	5M 3F	Pancreatic duct (8) • 8 benign (CP)	8	100	1 (12.5%) • 1 pancreatitis (mild) • No SAEs	75	2 (25%) remaining strictures at the 6 mo follow-up
Yue et al (15)	Case report	65	1M	Pancreatic duct (1) • 1 benign (CP)	1	100	0 (0%)	100	NA
Abou Zeid et al (16)	Case series	67	2M1F	Biliary duct (3) • 3 benign (2 cystic duct stricture during cholecystitis—1 inflammatory intrahepatic stricture)	3	100	0 (0%)	100	NA
Our series	Case series	66.88	6M 3F	Biliary stricture (8) • 4 benign (PO) • 4 malignant (CCK—2 liver metastasis—PDAC) Pancreatic duct (1) • 1 benign (CP)	9	100	1 (11%) • 1 minor hemobilia • No SAEs	89	• 1 early relapse (3 wk) • 1 remaining stricture at 6 mo • 1 remaining pain despite pancreatic calibration

CCK, cholangiocarcinoma; CP, chronic pancreatitis; NA, not available; PDAC, pancreatic ductal adenocarcinoma; PO, postoperative strictures; SAES, severe adverse event.

The mean age was 55 years (SD 12.2) among the 93 patients where it was reported. The population was predominantly men, with 66 men (65%) and 36 women (35%).

Biliary strictures were observed in 61 patients (60%), whereas 41 patients (40%) presented with pancreatic strictures. Among biliary indications, 44 were related to benign indications (72%), mostly after surgical complications (40/44). Seventeen patients (29%) had malignant biliary outlet obstruction, mostly related to liver metastasis or cholangiocarcinomas. All the pancreatic procedures were performed for chronic pancreatitis-related strictures of the main pancreatic duct.

### Case series from our center: patient characteristics

A description of our patients is presented in Table 2. Their mean age was 66.6 years (SD 12.19). Six patients were men and 3 women. Eight were biliary cases (7 malignant tumors and 1 benign anastomotic stricture), 1 patient had a main pancreatic duct stricture during chronic pancreatitis. Median follow-up time was 10 months (IQR 7–12). Figure 3 presents a case of benign biliary anastomotic stricture (case 8).

**Table 2.** Case series of our tertiary center

Patient	Indication	Procedure	Main outcome (technical success/adverse events)	Secondary outcome: clinical success	Secondary outcome: long-term outcome
Case 1 73 YO female	Hilar stricture (Bismuth IIIA) related to a cholangiocarcinoma developed over a primary sclerosing cholangitis.	Positioning of a hydrophile guidewire through a tight, and angulated stricture. Impossible to insert a balloon dilator. • Electrosurgical drilling. • Positioning of an OACL 10Fr × 15 cm stent.	Procedural success (technical success, no procedure related AEs).	Mild clinical success. Migration of the stent requiring a second procedure. Improvement after the second procedure.	No long-term clinical success. Relapse of the stricture 3 wk later. • Radiological external drainage. The patient died a month later after a surgical resection of the tumor.
Case 2 52 YO female	Metastatic meningioma with hepatic metastasis inducing a biliary dilatation of the right biliary ducts with a long and angulated stricture.	Positioning of an angulated guidewire but impossible to achieve dilation. • Electrosurgical drilling followed by a 4 mm dilation. • Insertion of an 18 cm, 8.5 Fr plastic stent (custom-made by cutting a nasobiliary drain).	Procedural success (technical success, no procedure-related AEs).	Clinical success Improvement of liver function tests.	Long-term clinical success No relapse of biliary obstruction during the following year. The patient died a year after, after a rapid progression of his meningioma.
Case 3 83 YO male	History of liver resection for metastatic squamous cells carcinoma. Biliary leak with hilar stricture. Repeated failure to drain the right posterior sector.	Positioning an angulated hydrophile guidewire across a long ischemic stricture. Impossibility do dilate. • Electrosurgical drilling followed by a 6 mm dilation. • Insertion of an 8.5Fr x 15 cm plastic stent.	Procedural success (technical success, no procedure related AEs).	Clinical success with improvement of liver function tests.	Loss of follow-up after the procedure.
Case 4 67 YO male	Liver transplant for hepatocarcinoma developed on an alcoholic cirrhosis. Complete anastomotic stricture.	Positioning of an hydrophile angulated guidewire. Impossibility to insert a dilation balloon through the stricture. • Electrosurgical drilling followed by a 6 mm dilation. • Insertion of a 10 mm × 8 cm fully covered metallic stent (FCSEMS).	Procedural success (technical success, no procedure related AEs).	Clinical success with improvement of the liver biology.	Long-term clinical success New ERCP 6 mo later with removal of the metallic stent. No remaining stricture. To date (2 yr from the procedure) no relapse of the stricture was noted with a tight follow-up.
Case 5 59 YO male	Liver transplant for a mixed alcoholic and metabolic liver disease. Early anastomotic stricture	Positioning of a guidewire through a short but tight and angulated stricture. Impossibility to insert a dilation balloon. • Electrosurgical drilling followed by a 6 mm dilation. • Insertion of a 10 mm × 8 cm FCSEMS.	Procedural success (technical success, no cystotome-related AEs). AE: Sphincterotomy-related hemobilia with spontaneous resolution.	Clinical success with improvement of the liver biology.	Mild long-term clinical success Refractory stricture with iterative changes of the metallic stent without complete calibration. The patient died 1 yr after the procedure due to a chronic rejection of the graft.
Case 6 50 YO male	Chronic pancreatitis. Biliary stricture treated with FCSEMS. Chronic pain with MPD dilation caused by calcifications. Two previous failures of MPD canulation.	Retrograde canulation of the main pancreatic duct was finally achieved with a hydrophile guidewire. Tight and short distal stricture uncrossable with the dilator. • Electrosurgical drilling followed by a 4 mm dilation. • Stenting with an 8.5Fr plastic stent.	Procedural success (technical success, no procedure related AEs).	Mild clinical success with only a slight pain improvement.	Mild long-term clinical success At 6 mo, removal of the stent. No remaining stricture but no real improvement of the patient's symptoms. No reintervention to date (16 mo follow-up).
Case 7 77 YO female	Pancreatic adenocarcinoma with duodenal stricture and distal biliary stricture. Choledocoduodenostomy with a LAMS inducing several reflux angiocholitis related to the duodenal stricture.	Rendezvous technique through the LAMS with insertion of a guidewire that passes through the biliary stricture into the duodenum. Duodenal stricture impassable with the endoscope but possibility to grasp the wire through the stricture. Owing to the positioning of the endoscope separated from the papilla by the stricture, a sphincterotomy was not possible under visual control and we failed to push a balloon dilator through the biliary stricture. • Electrosurgical drilling followed by an 8 mm dilation. • Stenting with a 6 cm × 10 mm FCSEMS covering the hole of the LAMS.	Procedural success (technical success, no procedure related AEs).	Clinical success Improvement of LFTs and possibility to resume chemotherapy.	Long-term clinical success Necessity to position a duodenal stent to compensate for increased duodenal tumor obstruction and deobstruction of the stent 3 mo after it’s positioning. To date (10 mo after the cystotome procedure), the patient remains stable on her liver biology and continues chemotherapy. No reintervention.
Case 8 78 YO male	Liver transplantation with anastomotic biliary stricture. Tight stricture with a first failed attempt to pass the stricture.	Successful crossing of the stricture with a guidewire. • Electrosurgical drilling through the stricture followed by 6 mm dilation. • Stenting with an 8 cm × 10 mm FCSEMS.	Procedural success (technical success, no procedure related AEs).	Clinical success With improvement of liver biology.	Long-term clinical success. Reintervention at 4 mo after the migration of the FCSEMS and reintervention at 7 mo after another migration with insertion of plastic stents (8-mo follow-up).
Case 9 78 YO male	Liver metastasis from a colorectal cancer. Previous right liver lobectomy bur relapse on the hilum. Patient already stented but persistence of a cholangitis with incomplete drainage of the left liver.	Complex hilar stricture. We manage to access segment III through a long and angulated stricture. Dilation failure. • Electrosurgical drilling followed by a 4 mm dilation and • Stenting with 2 uncovered metallic stents in segments II (10 cm × 10 mm) and III (12 cm × 10 mm).	Procedural success (technical success, no procedure related AEs).	Clinical success improvement of liver biology.	Long-term clinical success. No reintervention to date (7-mo follow-up).

AE, adverse event; ERCP, endoscopic retrograde cholangiopancreatography; FCSEMS, fully covered self expandable metallic stent; LAMS, lumen apposing metallic stent; LFTs, liver function tests; MPD, main pancreatic duct; NA, not available; OACL, OASIS stents.

**Figure 3.**
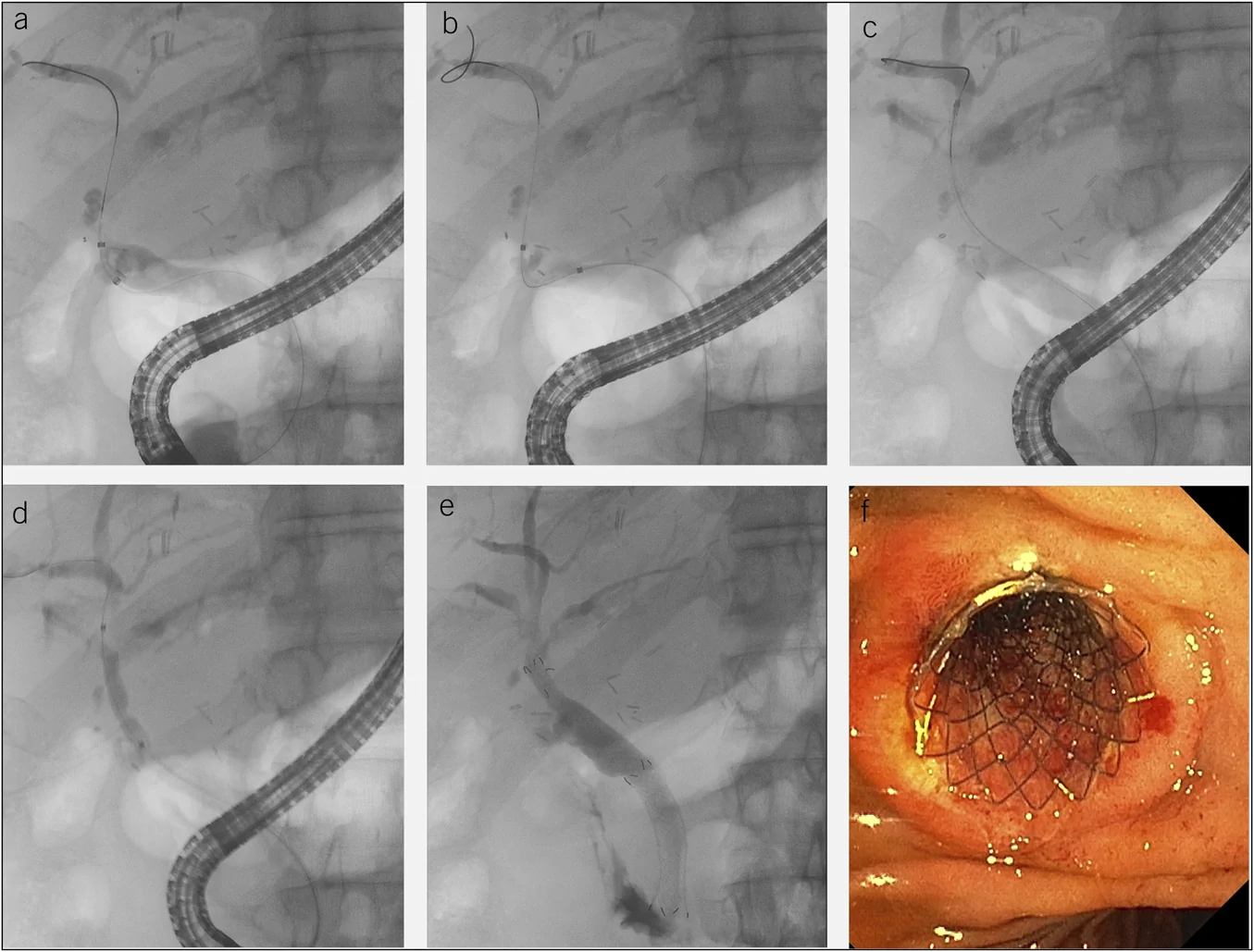
Illustrative case. (**a**) Passage of the stricture with a hydrophile guidewire. (**b**) Impossibility to progress with a balloon dilator. (**c**) Electrosurgical drilling with a 6FR cystotome. (**d**) 6 mm dilation of the stricture. (**e**) Stent deployment (fluoroscopic vision). (**f**) Stent deployment (endoscopic vision).

### Outcomes

The technical success rate, defined as a successful passage of a stricture with the cystotome and subsequent stenting was 100% (95% CI: 96.4–100) (102/102 patients). A total of 11 AEs were reported with 1 from our cohort being clearly sphincterotomy-, not cystotome-related—whereas no precision was provided for the other AEs. The cystotome-imputable AEs rate was therefore 9.8% (95% CI: 4.8–17.3). Overall, 90.2% of the population underwent a successful drainage with technical success without complication.

All complications were considered mild or moderate, with no case of perforation or severe (AGREE grade ≥3) adverse event observed. Among patients with pancreatic ERCP (N = 41), 3 mild pancreatitis (7.3%), 1 nonsevere bleeding (2.4%) and 3 transient abdominal pain (7.3%) episodes occurred. Among biliary ERCPs (N = 61), 1 cholangitis (1.6%) and 3 hemobilia (5.9%) cases occurred, 1 of the hemobilia cases from our cohort being observed during the sphincterotomy and before cystotome use regarding the causality of the cystotome. The incidence of the different reported complication is displayed in Figure 4.

**Figure 4.**
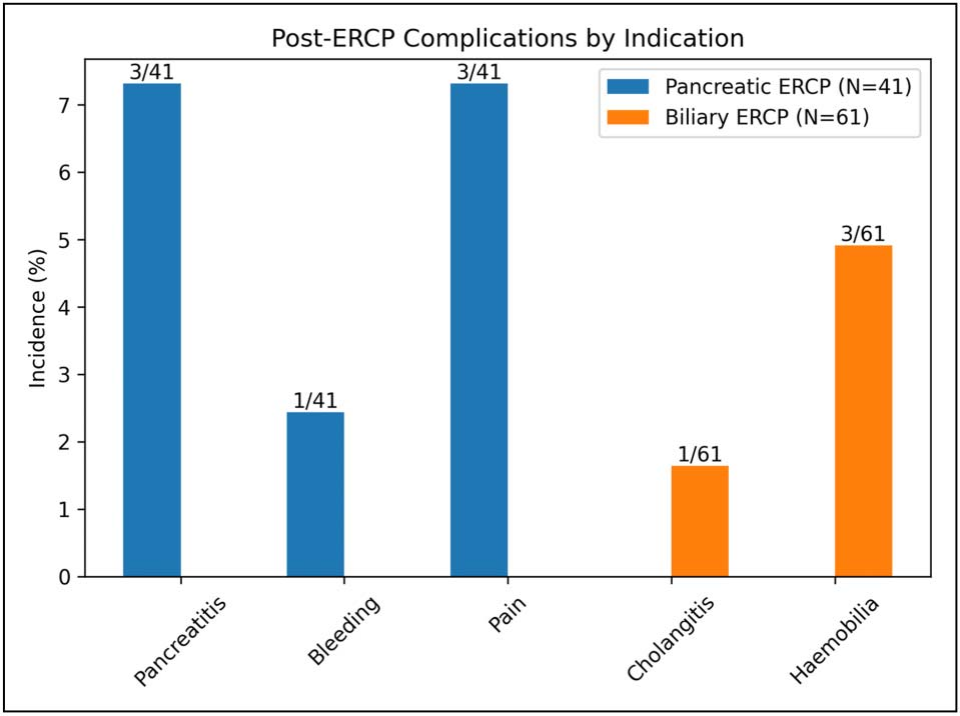
Incidence of the different complications. ERCP, endoscopic retrograde cholangiopancreatography.

Regarding secondary outcomes, clinical success was achieved in 84/89 patients (94%). The study by Kato et al (10) (13 patients) did not allow this analysis due to the absence of reported clinical outcomes and only long-term data on stent presence and stricture relapses.

Long-term outcomes were reported in 6 studies (67 patients—42 biliary strictures/25 pancreatic strictures). Twenty-six biliary strictures (62%) were resolved (no relapse or no remaining stricture after stent removal) after at least 3 months of follow-up. One pancreatic stricture was described as recurrent and at least 1 (from our cohort) of the 25 patients treated for a pancreatic stricture experienced no pain improvement after pancreatic calibration.

## DISCUSSION

This systematic review, complemented by a single-center case series, represents the first and most comprehensive synthesis to date of the efficacy and safety of cystotome-assisted electrosurgical recanalization in impassable biliopancreatic strictures. Although previous reports have presented this approach in anecdotal fashion, our study consolidates the evidence in a more cohesive assessment of its technical performance, clinical benefit, and associated adverse events (17).

Alternative rescue strategies have been described for completely impassable biliary strictures, including EUS-guided extra-anatomical drainage such as hepaticogastrostomy or other transluminal biliary anastomoses and percutaneous approaches. These techniques are particularly valuable when retrograde access cannot be achieved. However, in our series, most of the patients were treated for benign strictures, in whom the creation of a permanent extra-anatomical access route may not represent the optimal long-term solution. Moreover, cystotome-assisted drilling applies to a specific clinical scenario in which the stricture has already been contrast injected and successfully traversed with a guidewire, but in which conventional dilation devices fail to pass, preventing completion of stenting. In this setting, consolidating the existing transpapillary tract with a cystotome is technically straightforward and preserves the anatomical route. Switching to an extra-anatomical drainage strategy at this stage would result in loss of an already secured access and expose the patient to a more invasive procedure. It is to be noted that cystotome use increases procedural costs moderately (309€ Incl.Taxes) and are often available in the endoscopist's toolbox for therapeutic EUS procedures.

Various devices have been used in similar situations, including the Soehendra stent retriever who was originally designed to extract occluded plastic stents but proved to be better used as a worm gear, over-the-wire mechanical drill for otherwise impassable strictures. Case-series data attest its feasibility (11,18,19). Nevertheless, the stent retriever relies solely on mechanical torque without hemostatic effect thus potentially increasing the risk of bleeding. Combined with that, a rather unpractical handling led our team to give preference to the cystotome. Other teams reported the use of incisional devices such as wire-guided needle-knife (20). However, a guidewire-assisted needle knife in the bile duct is prone to carry a higher risk of biliary injury because the needle is not strictly coaxial, and it lacks the circumferential cut/coagulation effect achieved with a cystotome. Even more outlandish tools such as wire-guided biopsy forceps have been used to drill into pancreatic strictures. In 1 case series, the forceps progressed through the stricture partially opened with electrocautery current (20). These alternatives seem less precise and more hazard-prone than a dedicated wire-guided tool such as a cystotome. In 1 case report, a cystotome was used percutaneously without guide-wire guidance to open up a tight anastomotic stricture but it does not seem to us to be either an advisable or a reproductible strategy for bile duct strictures.

Another debatable topic is the applied current during cystotome-drilling. Alike most other authors, we used a sequential cut/coagulation mode, such as the one used during sphincterotomy, to secure slow-motion, bleed-preventing progression through the stricture. Some authors reported using pure-cut mode allowing faster passage through the stricture, but we believe that pure cut should be reserved for the toughest among tough cases or to avoid protracted cautery in the same area.

Our findings demonstrate an excellent technical success rate (100%) across all studies, confirming that the cystotome is an effective tool to overcome complex strictures that cannot be passed using conventional dilation techniques. In our cohort, we also used the cystotome in cases of difficult positioning with insufficient pushability to make instruments progress through the stricture. If this high success rate is impressive considering this technique as a rescue after previous failed attempts, it must be nuanced by the risk of publication biases in case series. In our experience as well, all procedures were technically successful, although performed in highly selected cases.

The overall cystotome-related adverse event rate was 9.8%. Given that technical success was achieved in 100% of procedures, the composite end point (technical success without adverse events) was reached in 90.2% of cases. Importantly, all adverse events were minor and did not compromise technical success. Hemorrhage, abdominal pain, acute pancreatitis and cholangitis, if reported in a few cases, were always treated conservatively and were never life-threatening or damaging to a major organ or vessel. Moreover, causality between the use of the cystotome and complication remains difficult to assess in this retrospective review as pancreatitis can be associated with pancreatic duct canulation itself and at least 1 of the bleeding cases (from our cohort) was clearly subsequent to the sphincterotomy.

We defined clinical success as the resolution of the condition that prompted the procedure, with stent placement being the usual therapeutic goal. Consequently, clinical success was not considered a primary end point because traversing the stricture was a prerequisite to achieve this goal, regardless of the method used. With this in mind, clinical success was also favorable, with 94% of immediate success among studies where it was reported.

Long-term outcomes were reported in some studies, but they are difficult to analyze or compare with the literature, as all procedures—whether biliary or pancreatic—were, by definition, complex, with the cystotome used as a rescue tool in heterogeneous indications including both malignant and benign diseases.

This review has several limitations. First, a formal meta-analysis was not performed due to the limited number of studies and the small sample sizes, which precluded robust statistical modeling and subgroup analysis. Second, there were no prospective trials available addressing this topic. Third, although the risk of bias was assessed using the JBI Critical Appraisal Checklist for Case Series which evaluates selection methods, measurement reliability, reporting transparency, and statistical appropriateness, our data originated from retrospective case series and reports with possible selection biases.

A center effect bias is likely, as most procedures were performed in expert centers by highly experienced endoscopists. The reproducibility of these results in community or lower-volume settings remains uncertain.

A publication bias cannot be excluded, as unsuccessful or complicated cases may be underreported in the literature, potentially leading to success overrating and underrating of complications.

In addition, there was significant heterogeneity in the definitions and timing of clinical success, ranging from early symptom relief to long-term radiologic or functional improvement. Moreover, it is difficult to compare clinical successes from different studies treating malignant/benign diseases from the biliary/pancreatic duct.

Nevertheless, our own center experience fits the literature results with a high efficiency and reasonable clinical outcome regarding the complexity of the cases with no additional adverse events compared with the expected complication rate of standard ERCP with sphincterotomy and stenting.

If we do not believe that cystotome-assisted electrocautery should become the first line therapy to overcome a stricture, nor that it offers longer biliary patency than alternative techniques, this study highlights its potential safety and efficacy and supports its use in trained centers after failure of conventional strategies.

However, as for clinical outcomes, a reliable assessment of adverse events should be confirmed by prospective studies.

## CONCLUSION

Cystotome-assisted electrosurgical drilling seems to be technically reliable and effective for complex biliopancreatic strictures that are resistant to standard dilation. We believe this comprehensive study should encourage seasoned endoscopists to integrate the cystotome in their ERCP toolbox for highly selected cases.

## CONFLICTS OF INTEREST

**Guarantor of the article: **Gabriel Marcellier, MD.

**Specific author contributions:** G.M.: data collection, literature review, data analysis, manuscript drafting. N.E.: data collection, literature review, data analysis, manuscript drafting. C.P., B.J., A.B., F.M., P.R.: endoscopic procedures, data interpretation, manuscript review. F.P.: study conception and design, methodology coordination, supervision, data interpretation, manuscript review.

**Financial support:** None to report.

**Potential competing interests:** None to report.

**Ethical statement:** No institutional review board (IRB) approval was required for this study. For the case series, informed non-opposition was obtained from all patients for the publication of anonymized data.

## Supplementary Material

## ABBREVIATIONS:

AEs: adverse events
ERCP: endoscopic retrograde cholangiopancreatography
EUS: endoscopic ultrasound
