# Supplementary material for: Cystotome Use for Impassable Biliopancreatic Strictures: Efficacy and Safety—Systematic Review and Single Center Case Series
Source: Clin Transl Gastroenterol. 2026 Mar 17;17(6):e01019. doi: 10.14309/ctg.0000000000001019 (PMC13641660; doi:10.14309/ctg.0000000000001019)
Supplement: Supplementary file 1 [file ct9-17-e01019-s001.pdf]

Supplementary Appendix 1 – Search string for each database

Database	PubMed	Cochrane	Google-Scholar	Embase
**Research Key-Words**	(cystotome[tiab] OR "diathermic dilator"[tiab] OR (diathermic[tiab] AND dilator*[tiab]) OR (wire-guided[tiab] AND (cystotome[tiab] OR diathermic[tiab])) OR electroincision[tiab]) **AND** (biliary[tiab] OR "bile duct"[tiab] OR pancreatic[tiab] OR "pancreatic duct"[tiab] OR biliopancreatic[tiab]) AND (stricture*[tiab] OR refractory[tiab] OR severe[tiab] OR tight[tiab] OR difficult[tiab] OR recalcitrant[tiab] OR recanalization[tiab] OR dilation[tiab] OR dilatation[tiab])	#1 cystotome:ti,ab,kw<br>#2 "diathermic dilator":ti,ab,kw<br>#3 #1 OR #2<br>#4 cystotome OR "diathermic dilator"<br>#5 #3 AND #4	"cystotome" / ”diathermic dilator” "biliary” / ”pancreatic” stricture" -EUS - pseudocyst	Cystotome AND biliary OR pancreatic strictures NOT EUS
**Search results**	**29**	**14**	**37**	**16**
**Included**	**7** (Kawakami 2014 – Puri 2018 - Kato 2019 – Rana 2021 – Sundaram 2021 – Tringali 2022 – Yue 2024)	**0**	**2** (Lee 2023 - Zeid 2025)	**0**
**Duplicate removal**	**0**	**0**	**14** (Kato 2019 – Kawakami 2014 – Sundaram 2021 – Rana 2019 – Ksissa 2025)	3 (Kato 2019 – Puri 2018 – Tringali 2022)
**Excluded**	**22**<br>- 15 (Out of subject)<br>- 1 Review Article (Ksissa 2025)<br>- 1 including only digestive strictures (Martinez 2017)<br>- 3 using devices other than cystotomes (Gao 2013 - Itoi 2010 – Yang 2015)<br>- 1 reporting a case report but followed by a case series by the same author with high risk of case duplicate (Kawakami H – 2012)<br>- 1 describing the use of a cystotome to repermeabilized a stent (Bronswijk M – 2019)	**14**<br>- 14 (Out of subject)	**35**<br>- 21 (Out of subject)<br>- 14 duplicate removal	**16**<br>- 11 (out of subject)<br>- 1 using a cystotome percutaneously “free hand” without guidewire guidance to reperabilize an anastomotic stricture (Drago 2025)<br>- 1 reporting a case series but followed by a case series by the same author a year after (Puri 2017)<br>- 3 duplicate removal

Supplementary Appendix 2 – Joanna Briggs Institute (JBI) Critical Appraisal Checklist

**QUESTIONS**	Were there clear criteria for inclusion in the case series?	Was the condition measured in a standard, reliable way for all participants included in the case series?	Were valid methods used for identification of the condition for all participants included in the case series?	Did the case series have consecutive inclusion of participants?	Did the case series have complete inclusion of participants?	Was there clear reporting of the demographics of the participants in the study?	Was there clear reporting of clinical information of the participants?	Were the outcomes or follow up results of cases clearly reported?	Was there clear reporting of the presenting site(s)/clinic(s) demographic information?	Was statistical analysis appropriate?	**Include / Exclude / Seek Further Info**
**Kawakami 2014**	**YES**	**YES**	**YES**	**YES**	**YES**	**YES**	**YES**	**YES**	**YES**	**YES**	**Include**
**Puri 2018**	**YES**	**YES**	**YES**	**YES**	**UC[1]**	**YES**	**YES**	**YES**	**YES**	**YES**	**Include**
**Kato 2019**	**YES**	**YES**	**YES**	**YES**	**YES**	**YES**	**YES**	**YES**	**YES**	**YES**	**Include**
**Rana 2021**	**YES**	**YES**	**YES**	**UC**	**UC**	**UC**	**YES**	**UC[2]**	**UC**	**YES**	**Include**
**Sundaram 2021**	**YES**	**YES**	**YES**	**UC**	**UC**	**YES**	**YES**	**YES**	**YES**	**YES**	**Include**
**Tringali 2022**	**UC[3]**	**YES**	**YES**	**NO**	**NO**	**YES**	**YES**	**YES**	**YES**	**NA**	**Include**
**Lee 2023**	**YES**	**YES**	**YES**	**YES**	**UC**	**UC**	**YES**	**UC[2]**	**YES**	**YES**	**Include**
**Yue 2024**	**YES[4]**	**YES**	**YES**	**NA**	**NA**	**YES**	**YES**	**YES**	**UC**	**NA**	**Include**
**Zeid 2025[5]**	**UC**	**UC**	**YES**	**UC**	**UC**	**YES**	**YES**	**UC**	**UC**	**UC**	**Include**

Yes – No – Unclear (UC) - Not Applicable (NA)

1) **Consecutive cases but no precision if all performed cases were included**
2) **Long term outcomes were not clearly reported**
3) **Describes “challenging strictures” as inclusion criteria without clear information about the attempts performed**
4) **Case report rather than case series**
5) **Abstract only, unclear for several parameters**
